# West Nile Virus Infection among Humans, Texas, USA, 2002–2011

**DOI:** 10.3201/eid1901.121135

**Published:** 2013-01

**Authors:** Melissa S. Nolan, Jim Schuermann, Kristy O. Murray

**Affiliations:** Author affiliations: Baylor College of Medicine, Houston, Texas, USA (M.S. Nolan, K.O. Murray);; Texas Department of State Health Services, Austin, Texas, USA (J. Schuermann)

**Keywords:** West Nile virus, Flavivirus, attack rates, relative risk, seroprevalence, economic cost, viruses, Texas

## Abstract

We conducted an epidemiologic analysis to document West Nile virus infections among humans in Texas, USA, during 2002–2011. West Nile virus has become endemic to Texas; the number of reported cases increased every 3 years. Risk for infection was greatest in rural northwestern Texas, where *Culex tarsalis* mosquitoes are the predominant mosquito species.

The first documented case of West Nile virus (WNV) infection in North America occurred during an outbreak of encephalitis in 1999 in New York, New York, USA (1). Within a few years, WNV had rapidly spread across the United States and was being transmitted throughout most of the country (2). In Texas, the first cases of human infection with WNV were reported in 2002, and cases have been reported every year since. Our objective was to epidemiologically describe WNV infections among humans over the first decade of virus transmission in Texas.

## The Study

We analyzed deidentified surveillance data for all cases reported to the Texas Department of State Health Services (TxDSHS) during 2002–2011. Reporting of West Nile neuroinvasive disease (WNND) to the TxDSHS was made mandatory in 2000, and West Nile fever (WNF) was added to the list of reportable conditions in 2005. Onset dates were categorized according to work week for each year and were used to create an epidemic curve and yearly incidence graph. Attack rates (no. cases/100,000 population) were stratified by demographics, and a 2-tailed *t* test was used to detect differences between the overall attack rate for the state and each demographic variable. Stratified calculations were based on the number of variable-specific reported cases over the variable-specific population. We estimated the seroprevalence by using published ratios (no. infected: no. WNND cases) and state population estimates for 2011 (3–5). By using the number of reported WNND cases, defining WNF as 26% of seroprevalence estimates, and using previously published cost estimates for WNND and WNF cases, we calculated the total economic cost of infections over the past decade (6,7).

Relative risks (RRs) for each county were calculated for each year. Expected number of cases per county (*e*) were calculated by using a validated equation *e* = (*r* × *n*) (*c*), where *r* is the state incidence rate, *n* is the state population, and *c* is the county population (5,8). To assess potential associations between counties and increased RRs and to examine urban–rural characteristics of counties and increased RRs, we performed Kruskal-Wallis 1-way analysis of variance by ranks. All calculations were run by using Stata version 12.0 software (StataCorp, College Station, TX, USA).

**Figure 1:**
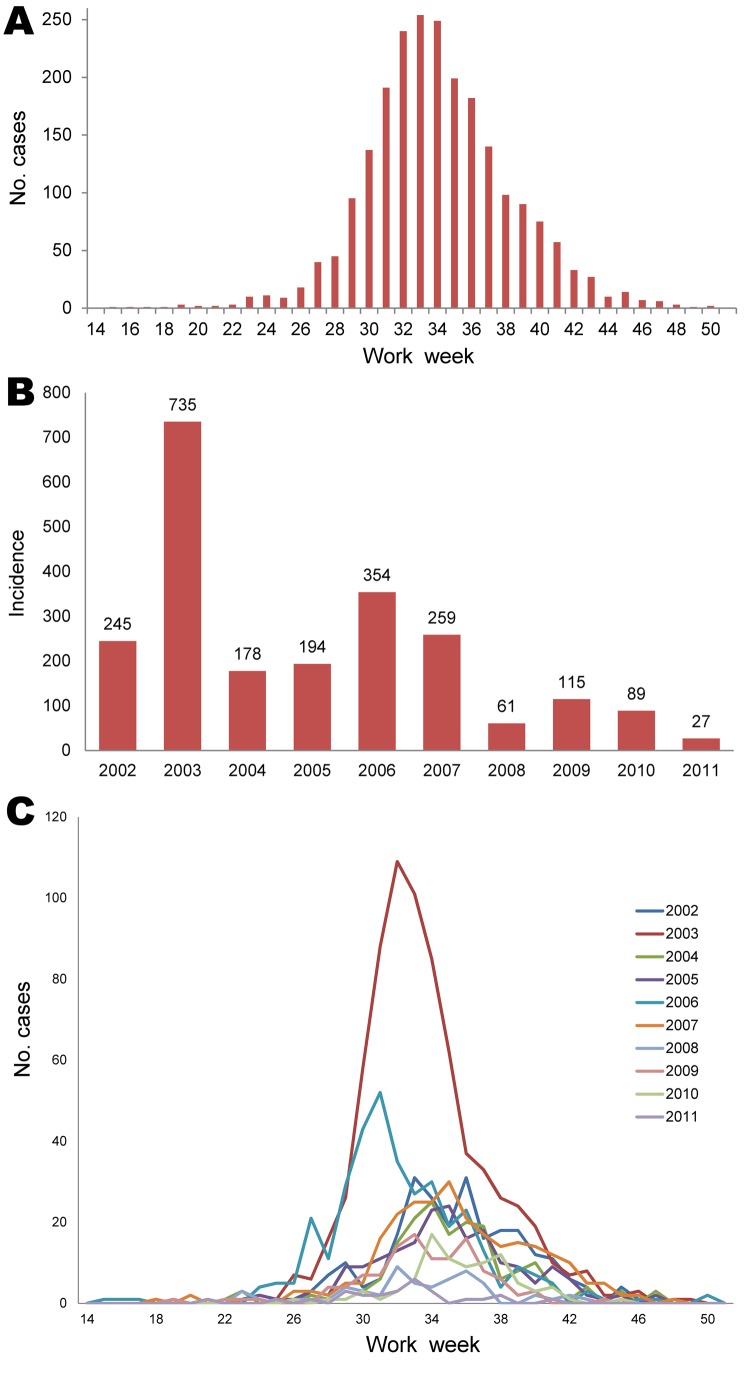
Reported cases of West Nile virus infection among humans, Texas, USA, 2002–2011. A) Epidemic curve. B) Incidence (no. cases/100,000 population). C) Epidemic curve line graph.

During 2002–2011, a total of 2,274 cases were reported to TxDSHS. Most (n = 735) cases were reported in 2003, and additional peaks occurred in 2006 (n = 354) and 2009 (n = 115). These peaks occurred every 3 years, starting in 2003, with a higher number of cases reported in those peak years than in the years before and after (Figure 1). Transmission season in Texas was April–December; the epidemic curve peaked in August.

Of those cases reported, 749 (33%) were WNF and 1,525 (67%) were WNND. Most cases were in non-Hispanic white persons (1,224 [54%] cases), followed by Hispanic (550 [24%]), other/unknown (350 [15%]), non-Hispanic black (141 [6%]), and Asian (9 [1%]) persons. The median age of case-patients was 54 years (range 21 days through 99 years), and most (1,335 [59%]) were male. Case-fatality rate was 6.3% (143 deaths/2,274 cases); all deaths were attributed to WNND.

We calculated the attack rates for WNV for Texas, stratified by demographic variables (Table). The overall attack rate was 7.1 WNV cases per 100,000 population. Attack rates were higher among male than among female persons (10.2 cases and 6.7 cases/100,000 population, respectively), and attack rates among non-Hispanic white persons were slightly higher than those among persons of other races (11 and 8 cases/100,000 population, respectively). No statistical differences were found between the state overall attack rate and attack rates by sex or race/ethnicity. Our findings contradict those of previous studies that found higher attack rates for persons who were male or members of a minority group (3,9,10). Analysis of these variables on a smaller geographic level might provide more insight into the influence of demographics and socioeconomic status on infection rates.

**Table:** Sex- and age­­­-stratified ratio of infected population by reported cases of WNND, Texas, USA, 2002–2011*

Patient age, y	Texas population†	Reported WNND cases‡	WNND attack rate/100,000 population	Ratio of estimated no. infected for each reported WNND case§	Estimated WNV-infected population	Estimated seroprevalence of WNV- infected population, %
Children, 5–15	3,754,316	40	1.1	4,167	166,680	4.44
Adult men						
16–24	1,502,524	48	3.2	719	34,512	2.30
25–44	3,204,788	167	5.2	356	59,452	1.86
45–64	2,839,796	310	10.9	248	76,880	2.71
>65	1,098,089	358	32.6	50	17,900	1.63
Adult women						
16–24	1,442,664	37	2.6	1,231	45,547	3.16
25–44	3,066,278	140	4.6	330	46,200	1.51
45–64	2,920,842	190	6.5	387	73,530	2.52
>65	1,413,691	224	15.8	61	13,664	0.97
Overall	21,242,988	1,514	7.1	353	534,442	2.52

*WNND, West Nile neuroinvasive disease; WNV, West Nile virus. †Eestimates from State Data Center (www.idserportal.utsa.edu/sdc/projections).  ‡Reported to the Texas Department of State Health Services.  §Ratio from references (3,4)*.*

Attack rates for WNV increased progressively with age; the rate among persons >65 years of age was 23× higher than that among children 5–15 years of age. These findings are consistent with those of studies that found that WNV disease severity increases with age, resulting in a higher risk for disease among elderly persons (3,4,11).

By using published seroprevalence estimates of the ratio of asymptomatic blood donors to reported WNND cases, we were able to estimate that ≈534,000 persons in Texas have been infected with WNV since 2002 (Table). Using these numbers, we estimated an overall WNV seroprevalence of 2.5% and an estimated total economic cost of acute WNV infection in Texas of ≈$112 million. WNF cases cost $42 million ($302/case), and WNND cases cost $70 million ($46,530/case).

For the 10-year period, attack rates were calculated per county. Rates (no. cases/100,000 population) were highest for predominately rural counties within the northwest area of the state: Castro (34.2), King (27.9), Crosby (21.3), Swisher (18.7), and Parmer (16.4) Counties. Attack rates were low for the most densely populated counties; rates for the 5 metropolitan counties were as follows: Dallas (1.1), Tarrant (1.0), Harris (1.0), Travis (0.5), and Bexar (0.2) Counties.

**Figure 2:**
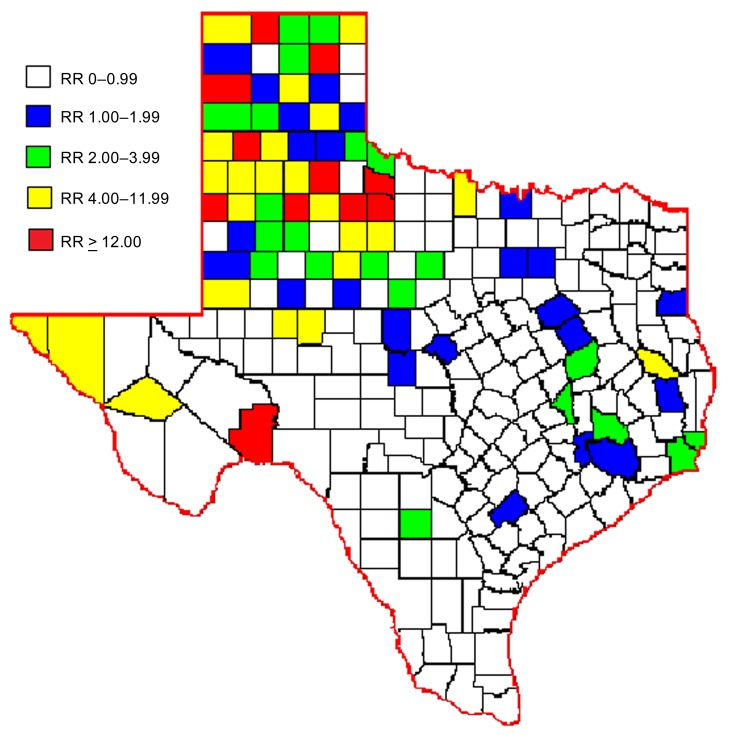
Average relative risk (RR) for human infection with West Nile virus, by county, Texas, USA, 2002–2011.

Over the 10 years, average RR was greater than expected (RR>1) for 79 (31%) of the 254 counties. The 10-year average RR was mapped by county (Figure 2). After mapping the RRs, we noticed spatial clustering and examined RR by region as defined by the Texas Association of Regional Councils (12). Analysis of variance indicated a significant difference of RR (p<0.001) by region. Odds of WNV infection in humans were significantly higher for 3 regions (ORs 23.6, 37.48, 2.7) in the northwestern part of the state than for other regions.

To further investigate RRs and environmental factors, we examined the percentage of urban versus rural land use in each county. Increased RR was positively associated (p = 0.01) with rural environments; median percentage of rural land use was 53% for rural counties with RR<3, compared with 85% for rural counties with RR>3.

We propose that predominant mosquito species combined with urban versus rural factors might have influenced disease transmission in Texas. Population densities varied; urban areas were generally in the eastern part of the state and rural areas in the western part (5). *Culex quinquefasciatus* mosquitoes are the predominant species in eastern Texas and are associated with urban areas; *C. tarsalis* mosquitoes are predominant in western Texas and are associated with rural areas (13–15). Unfortunately, reporting of WNV-positive mosquito species to TxDSHS is voluntary and was incomplete because of inadequate resources for surveillance in most Texas counties. This lack of mosquito data prohibited us from statistically evaluating the influence of *C. tarsalis* mosquitoes on WNV transmission to humans. According to the literature and our preliminary data, we believe *C. tarsalis* mosquitoes might be a factor associated with increased RR in western compared with eastern Texas, where *C. quinquefasciatis* mosquitoes are the more established species.

## Conclusions

During the 10 years of WNV emergence and human infection in Texas, a total of 2,274 cases were reported (67% WNND, 33% WNF); case-fatality rate was 6%. The total economic toll of human disease in Texas was $112 million. Although attack rates and RRs were higher for persons in rural counties, specifically in northwestern Texas, the highest numbers of cases were reported from metropolitan cities.
